# Editorial: Community series in mouse models of B cell malignancies, volume II

**DOI:** 10.3389/fimmu.2024.1488601

**Published:** 2024-09-23

**Authors:** Gema Perez-Chacon, Christelle Vincent-Fabert, Juan M. Zapata

**Affiliations:** ^1^ Instituto de Investigaciones Biomédicas Sols-Morreale CSIC-UAM, Madrid, Spain; ^2^ Instituto de Investigación Sanitaria La Paz (IdIPAZ), Madrid, Spain; ^3^ UMR CNRS 7276/INSERM U1262 CRIBL, University of Limoges, Limoges, France; ^4^ Hematology Laboratory of Dupuytren, Hospital University Center (CHU) of Limoges, Limoges, France

**Keywords:** genetically engineered mouse models, GEMM, B cell malignancies, mouse lymphoma, mouse leukemia, B cell neoplasms, CLL, DLBCL

B-cell malignancies are a diverse group of cancers that originate from B lymphocytes at different developmental and differentiation stages exhibiting distinctive progression patterns and symptoms. Prognosis depends on the histological type, disease stage, and available treatment (reviewed in (1–3). In the last decades, advances in medical research have significantly improved the outcomes for these patients. However, a deeper knowledge on the origin, progression, and development of refractoriness and relapse is needed for providing patients with new treatments with better remission rates or longer progression-free survival. In addition, tools allowing a more accurate preclinical prediction on how new therapeutic approaches would work in clinics are also needed.

In this regard, mouse models of B cell malignancies are still indispensable for advancing our knowledge of B-cell hematological diseases. These models allow researchers to study the development, progression, and treatment of diseases in a controlled environment similar to that of the patient. Mice can be genetically modified to mimic human diseases, providing valuable information about the genetic factors involved in B-cell malignancies. Additionally, they help to understand the role of the immune system in these diseases, including how B cells interact with other immune cells and with the microenvironment cells and stroma. They are also important tools to test new therapies before clinical trials, ensuring safety and efficacy, as well as to reveal the underlying mechanisms of disease progression and treatment resistance.

In this Volume 2, three reviews and one perspective addressing mouse models of leukemia and lymphomas are included, providing a valuable extension to the Volume 1 of the collection (ISSN 1664-8714).

Bertilaccio and Chen contribute an interesting viewpoint on several genetic engineered mouse models (GEMMs) and patient-derived xenografts (PDXs) of chronic lymphocytic leukemia (CLL) and Richter’s syndrome (RS), pointing out the difficulty to obtain models recapitulating the high variability observed in CLL patients. Also, they reflect the importance of the tumor microenvironment in the maintenance and proliferation of CLL cells and how this can affect the progression into more aggressive forms such as RS and diffuse large B cell lymphoma (DLBCL)-RS.

Also focused on leukemia, Casado-García et al. follow the “two-step” model of B-cell acute lymphoblastic leukemia (B-ALL) proposed by Greaves (4) to discuss about the genetic and environmental factors leading to childhood B-ALL. They also review the B-ALL mouse models available to study this disease focusing on the “two-step” model. B-ALL development requires an initial genetic alteration to produce preleukemic clones already in uterus. However, only a percentage of children in this preleukemic state will develop B-ALL after exposing to certain environmental factors, such as infections, ion radiations or gut microbiome alterations. The authors highlight the difficulty of introducing a controlled environmental risk factor that reproducibly triggers childhood B-ALL in the available mouse models of this disease.

Tabatabai et al. present an in-depth and comprehensive review recapitulating various genetic mouse models of DLBCL. DLBCL is a highly heterogeneous hematological disease that has been recently divided into 5 clusters based on genomic analyses (5). Authors provide a detailed review attending to this classification. They discuss the distinct mutations characteristic of each cluster and the existing mouse models of each type. They propose several innovative ideas for future models that could more accurately capture the heterogeneity of this disease.

Finally, Li et al. present a perspective based on their previous research (6) on lymphoma dissemination in mouse models of B-cell aggressive lymphomas. They suggest that extranodal spread of lymphoma is the result of lymphoma cells losing their homing ability to lymphoid organs, rather than gaining the ability to target non-lymphoid organs. This hypothesis is based on the identification by the authors of mouse lymphoma cell lines stablished from mouse models of lymphoma that, once transplanted to mice, fail to migrate to lymphoid nodes and are only able to metastasize to distinct non-lymphoid organs (6).

In summary, the articles in this volume 2 provide a deep and updated review on the pathophysiology of CLL, DLBCL and B-ALL and a challenging perspective on the mechanism of lymphoma dissemination. Overall, these articles highlight the essential role of genetically modified mice in understanding all aspects of B cell lymphoma and leukemia.
